# Identification and potential mechanisms of a 4-lncRNA signature that predicts prognosis in patients with laryngeal cancer

**DOI:** 10.1186/s40246-019-0230-6

**Published:** 2019-08-15

**Authors:** Guihai Zhang, Erxi Fan, Qiuyue Zhong, Guangyong Feng, Yu Shuai, Mingna Wu, Qiying Chen, Xiaoxia Gou

**Affiliations:** 1 Department of Head and Neck Oncology, Affiliated Hospital of Zunyi Medical University, Zunyi, 563000 Guizhou Province People’s Republic of China; 2 Zunyi Medical University, Zunyi, 563000 Guizhou Province People’s Republic of China

**Keywords:** Long noncoding RNA, Laryngeal cancer, TCGA, Prognosis signature, Risk score

## Abstract

**Purpose:**

This study aimed to describe the use of a novel 4-lncRNA signature to predict prognosis in patients with laryngeal cancer and to explore its possible mechanisms.

**Methods:**

We identified lncRNAs that were differentially expressed between 111 tumor tissue samples and 12 matched normal tissue samples from The Cancer Genome Atlas Database (TCGA). We used Cox regression analysis to identify lncRNAs that were correlated with prognosis. A 4-lncRNA signature was developed to predict the prognosis of patients with laryngeal cancer. The receiver operating characteristic (ROC) curves and area under the curve (AUC) were used to verify the validity of this Cox regression model, and an independent prognosis analysis was used to confirm that the 4-lncRNA signature was an independent prognostic factor. Furthermore, the function of these lncRNAs was inferred using related gene prediction and Gene ontology (GO) enrichment analysis in order to clarify the possible mechanisms underlying their predictive ability.

**Results:**

In total, 214 differentially expressed lncRNAs were identified, and a 4-lncRNA signature was constructed using Cox survival analysis. The risk coefficients in the multivariate Cox analysis revealed that LINC02154 and MNX1-AS1 are risk factors for laryngeal cancer, whereas MYHAS and LINC01281 appear to be protective factors. The results of a functional annotation analysis suggested that the mechanisms by which these lncRNAs influence prognosis in laryngeal cancer may involve the extracellular exosome, the Notch signaling pathway, voltage-gated calcium channels, and the Wnt signaling pathway.

**Conclusion:**

We identified a novel 4-lncRNA signature that can predict the prognosis of patients with laryngeal cancer and that may influence the prognosis of laryngeal cancer by regulating immunity, tumor apoptosis, metastasis, invasion, and other characteristics through the Notch signaling pathway, voltage-gated calcium channels, and the Wnt signaling pathway.

**Electronic supplementary material:**

The online version of this article (10.1186/s40246-019-0230-6) contains supplementary material, which is available to authorized users.

## Introduction

Laryngeal cancer is one of the most common malignant head and neck tumors, with an estimated incidence of approximately 11,150 new cases in the United States in 2018, resulting in 3710 deaths. This cancer accounts for approximately 0.8% of all new cancer cases, and the mortality rate is nearly 0.6%. Accumulating studies suggest that the main risk factors for laryngeal cancer include tobacco, human papillomavirus infection, laryngopharyngeal reflux, environmental and occupational exposures, and alcohol [1]. Over the past few decades, various strategies have been employed to treat laryngeal cancer, but the prognosis of this disease remains unsatisfactory for patients and physicians [2]. According to 2006–2012 data from the SEER database, the 5-year OS of patients with laryngeal cancer remains as low as 60.7%, with no obvious improvement during the past several decades.

Presently, treatment strategies for laryngeal cancer mainly involve surgery and radiation therapy, as well as chemotherapy and gene therapy. Clinical relapse and poor prognosis are most common in cases of advanced laryngeal cancer with local invasion and distant metastasis. When laryngeal cancer is diagnosed at an early stage, the cure rate can reach 80% ~ 90% [3]. However, a majority of patients are still diagnosed with locally advanced disease or regional nodal metastases, and their survival rates are generally less than 50% [4–6]. Therefore, there is an urgent need to explore novel indicator for tumor detection with prognostic value.

With the development of molecular biology, increasingly more researchers have begun to investigate methods that could be used to predict the prognosis of laryngeal cancer and develop novel therapeutic strategies. lncRNAs are a class of RNAs that are usually more than 200 nucleotides in length and do not code for proteins [7]. Previous reports have shown that lncRNAs can modulate carcinogenesis and influence the rates of metastasis and invasion in various types of cancer [8–11]. Thus, the potential role and prognostic value of many lncRNAs needs to be further explored.

In our study, we used Cox regression survival analysis to construct a novel 4-lncRNA signature that is predictive of prognosis in laryngeal cancer. The final Cox regression model could be applied in clinical settings to predict the prognosis of patients with laryngeal cancer.

## Methods

### Workflow

**Fig. 1:**
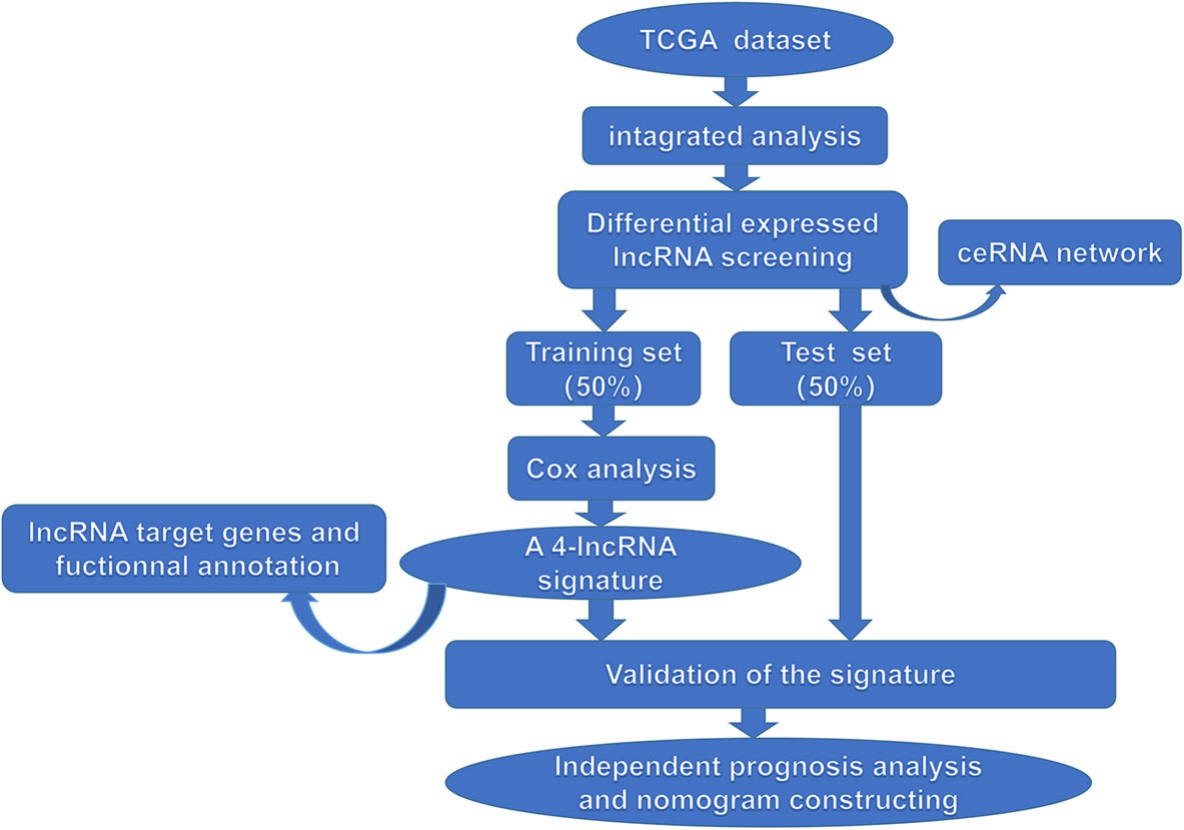
Main workflow for the study

We used a combination of methods in several steps to develop the 4-lncRNA signature and explore the possible mechanisms by which these lncRNAs influence the prognosis of laryngeal cancer (Fig. 1).

### Dataset processing and screening of differentially expressed lncRNAs

We collected samples and clinical materials from the TCGA (https://portal.gdc.cancer.gov/) and selected the HTseq-count. Differentially expressed lncRNAs were selected using edgeR software (*P*-value ≤0.001 and fold change ≥2) [12].

### ceRNA network construction and KEGG enrichment

We used the miRcode database (Jeggari, Marks & Larsson 2012) to predict lncRNA-miRNA interactions. The corresponding coding genes were then identified using miRDB, miRTarBase, and TargetScan [13–15]. We only chose miRNA-targeted mRNAs present in all three databases and differentially expressed mRNAs to enhance the validity of this ceRNA network. Furthermore, the ceRNA network was visualized using Cytoscape v3.6.1 [16].

### lncRNA signature identification and risk score calculation

To identify a multi-lncRNA signature with good predictive performance for prognosis, we randomly divided the samples into a training set and a test set. Differentially expressed lncRNAs that were correlated with patients’ OS in the training set were screened using univariate Cox regression analysis. lncRNAs with a *P* value < 0.05 were considered as candidate variables, and the lncRNAs with the lowest Akaike information criterion (AIC) values were retained in the final signature. The risk coefficients for these lncRNAs were calculated using a multivariable Cox proportional hazards model implemented with the “survival” and “survminer” R packages. Detailed R code is presented in the Additional file 2. We used the following risk formula: *riskscore* =  ∑ *β* ln c*RNA*i × *E*xp ln c*RNA*i (i = 1-n), where *β*_lncRNAi_ indicates the *coef* and Exp_lncRNAi_ indicates the expression level in patients with laryngeal cancer. All patients were divided into high-risk and low-risk groups according to their median risk score. We used the Kaplan–Meier method to analyze the OS of the two groups and the prediction value of the model was verified by plotting receiver operating characteristic curves (ROCs) for the training set, the test set, and the entire set. All data were processed and analyzed using R (version 3.5.1) and Perl 5 version 24 [17].

### Independent prognosis analysis and nomogram construction

The prognostic value of clinical variables and the risk score calculated using the lncRNA signature for the OS of laryngeal patients was initially assessed in univariate Cox proportional hazards regression analyses. Subsequently, each variable was evaluated in a multivariate Cox proportional hazards regression analysis. Furthermore, a nomogram to predict patients’ prognosis was constructed using R packages “Hmisc”, “lattice”, “Formula”, “ggplot2”, “foreign” and “rms”.

### Prediction analysis of lncRNA-related genes and gene ontology (GO) functional annotation

We identified genes related to the lncRNAs in the predictive signature using the Multi Experiment Matrix (MEM) database (https://biit.cs.ut.ee/mem/). We then uploaded these genes to the David website (https://david.ncifcrf.gov) to perform GO functional enrichment analysis, with *P* < 0.05 as the cutoff value. Finally, the results were visualized using Cytoscape v3.6.1 [16].

## Results

### Characteristics of the datasets

We collected 123 samples from 112 patients with laryngeal carcinoma from the TCGA, consisting of 111 laryngeal tumor tissue samples and 12 matched normal tissue samples. The clinical data and materials were collected from 92 men and 20 women, of whom 43 had died. The age of patients with laryngeal cancer ranged from 38 to 83 years, and their survival post-diagnosis ranged from 2 to 6417 days. More specific characteristics are shown in Table 1.

**Table 1:** Characteristics of the datasets

Variable	Case, *n*
Age	
≤ 49	11
>49	101
Gender	
male	92
female	20
T stage	
T1 + T2	20
T3 + T4	79
TX	11
NA	2
Lymph node status	
N0	40
N1–3	53
NX	17
NA	2
Metastasis	
M0	41
M1	1
MX	8
NA	62

### Differential expression of lncRNAs

**Fig. 2:**
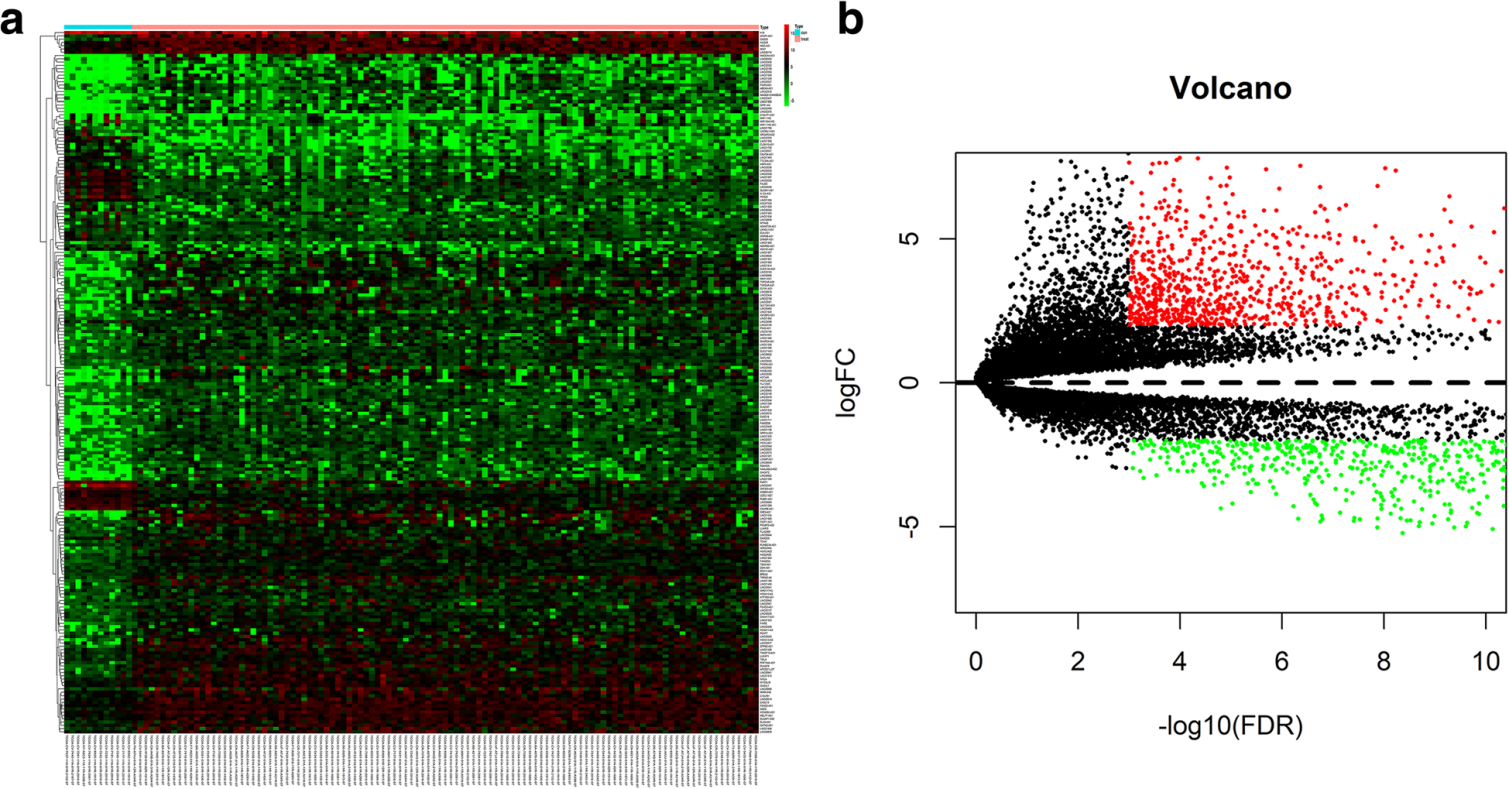
**a** Heatmap of differentially expressed lncRNAs. We conducted gene expression profiling to identify lncRNAs that were differentially expressed between 111 tumor tissue samples and 12 normal tissue samples. **b** Volcano plot of differentially expressed lncRNAs. Each red dot represents an upregulated gene, and each green dot represents a downregulated gene

A total of 214 differentially expressed lncRNAs were screened from the TCGA datasets (fold-change ≥2, *P* < 0.001), including 54 downregulated lncRNAs and 160 upregulated lncRNAs. Figure 2 illustrates the upregulated and downregulated genes using a volcano map and a heatmap.

### ceRNA network construction and KEGG enrichment analysis

We used 214 differentially expressed lncRNAs retrieved from the mircode database and identified 1597 pairs of interacting lncRNAs and miRNAs using the Perl program. In all, we identified a total of 28 lncRNA nodes, 18 miRNA nodes and 40 mRNA nodes as differentially expressed profiles in the ceRNA network (Additional file 1), but none of the plausible ceRNAs were associated with laryngeal cancer survival.

### Identification of a 4-lncRNA signature

**Fig. 3:**
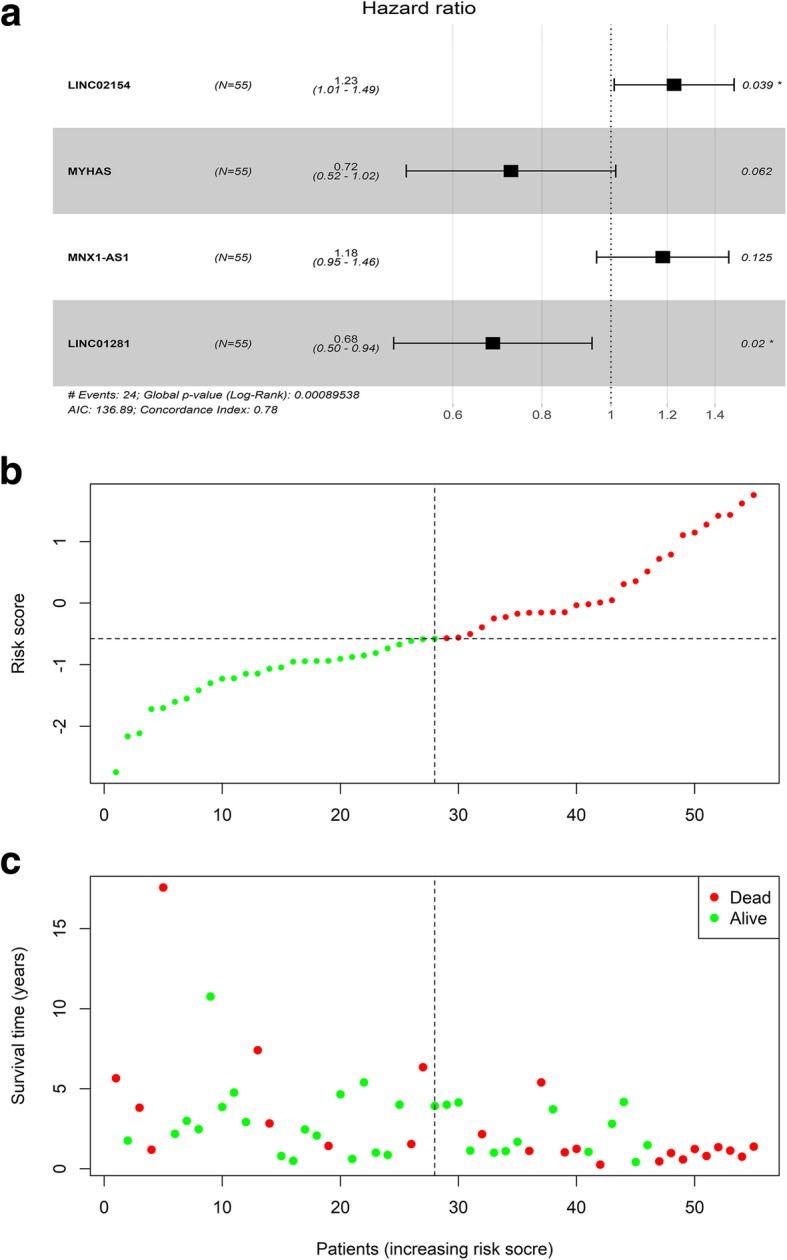
**a** Forest map of the 4-lncRNA signature. **b** lncRNA risk score. **c** Distribution of patient survival status and survival time

We randomly divided 110 samples and the corresponding clinical data into a training set (*n* = 55) and a test set (n = 55). In the training set, 214 differentially expressed lncRNAs were screened using univariate survival analysis to identify those that best correlated with prognosis. Multivariate Cox regression analysis was then used to develop a 4-lncRNA signature that is an independent predictor of 5-year OS (*P* < 0.05). The 4 lncRNAs included in this signature were LINC02154, LINC01281, MYHAS, and MNX1-AS1. The risk coefficients suggested that LINC02154 and MNX1-AS1 are risk factors for laryngeal cancer (*coef* > 0), whereas MYHAS and LINC01281 appeared to be protective factors (*coef* < 0) (Table 2). This 4-lncRNA signature is of great importance for prognostic evaluation in laryngeal cancer. The risk score of each patient was calculated based on the following formula: *riskscore* =  ∑ *β* ln c*RNA*i × *E*xp ln c*RNA*i (i = 1–4) = 0.204491007100773 × *E*xp_LINC02154_ + − 0.32288924590121 × Exp_MYHAS_ + 0.167587835674659× *E*xp_MNX1-AS1_ + − 0.381674952789156 × *E*xp_LINC02181_. Higher risk scores represent worse clinical prognosis. Accordingly, we divided patients into high- and low-risk groups depending on their median risk score to assess the score’s ability to accurately predict survival in a Cox regression model. The survival curve revealed a significant difference in OS between the high- and low-risk groups, confirming that the risk score could be an independent predictor of OS (Fig. 3).

**Table 2:** A 4-lncRNA signature identified by multivariate Cox regression analysis

id	coef	exp (coef)	se (coef)	z	Pr(>|z|)
LINC02154	0.204491007	1.226900422	0.098957801	2.066446566	0.038786333
MYHAS	−0.322889246	0.724054042	0.172769207	−1.868905064	0.061636024
MNX1-AS1	0.167587836	1.182449147	0.109102613	1.536057025	0.124524395
LINC01281	−0.381674953	0.682716932	0.163786433	−2.330320927	0.019789195

### Validation of cox regression model

**Fig. 4:**
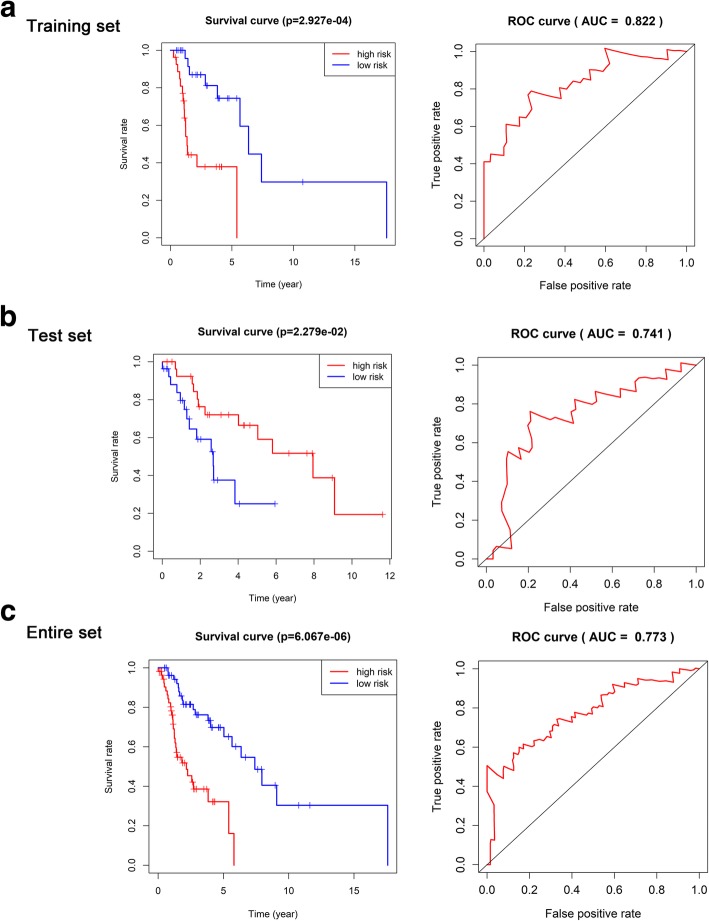
Validation of the 4-lncRNA signature. The area under the ROC was 0.822 in the training set, 0.741 in the test set, and 0.773 in the entire set. There were significant differences in OS between the high- and low-risk groups in all three sets. The results show that the 4-lncRNA signature performed well in predicting the prognosis of laryngeal cancer. **a** Training set, **b** Test set, **c** Entire set

**Fig. 5:**
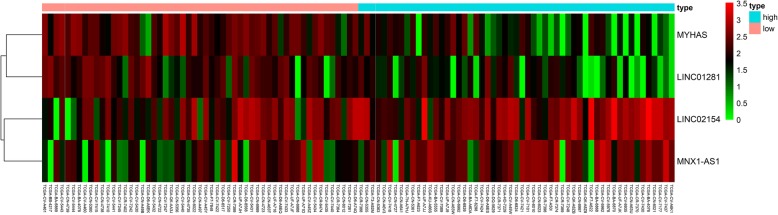
Heatmap of the 4-lncRNA signature in the TCGA datasets. Each column represents a sample, and each row represents one of the 4 lncRNAs. The expression levels of the 4 lncRNAs are shown in different colors, varying from orange to blue with increasing risk

The AUC of the ROC curve was 0.822 in the training set, 0.741 in the test set, and 0.773 in the entire set. Moreover, there were significant differences in OS between the high- and low-risk groups in all three sets, demonstrating that this signature performs well as a predictor of prognosis in laryngeal cancer patients (Fig. 4). Figure 5 shows a heatmap illustrating the distribution of risk scores across all patients for each gene.

### The 4-lncRNA signature is an independent predictor of laryngeal cancer prognosis

**Fig. 6:**
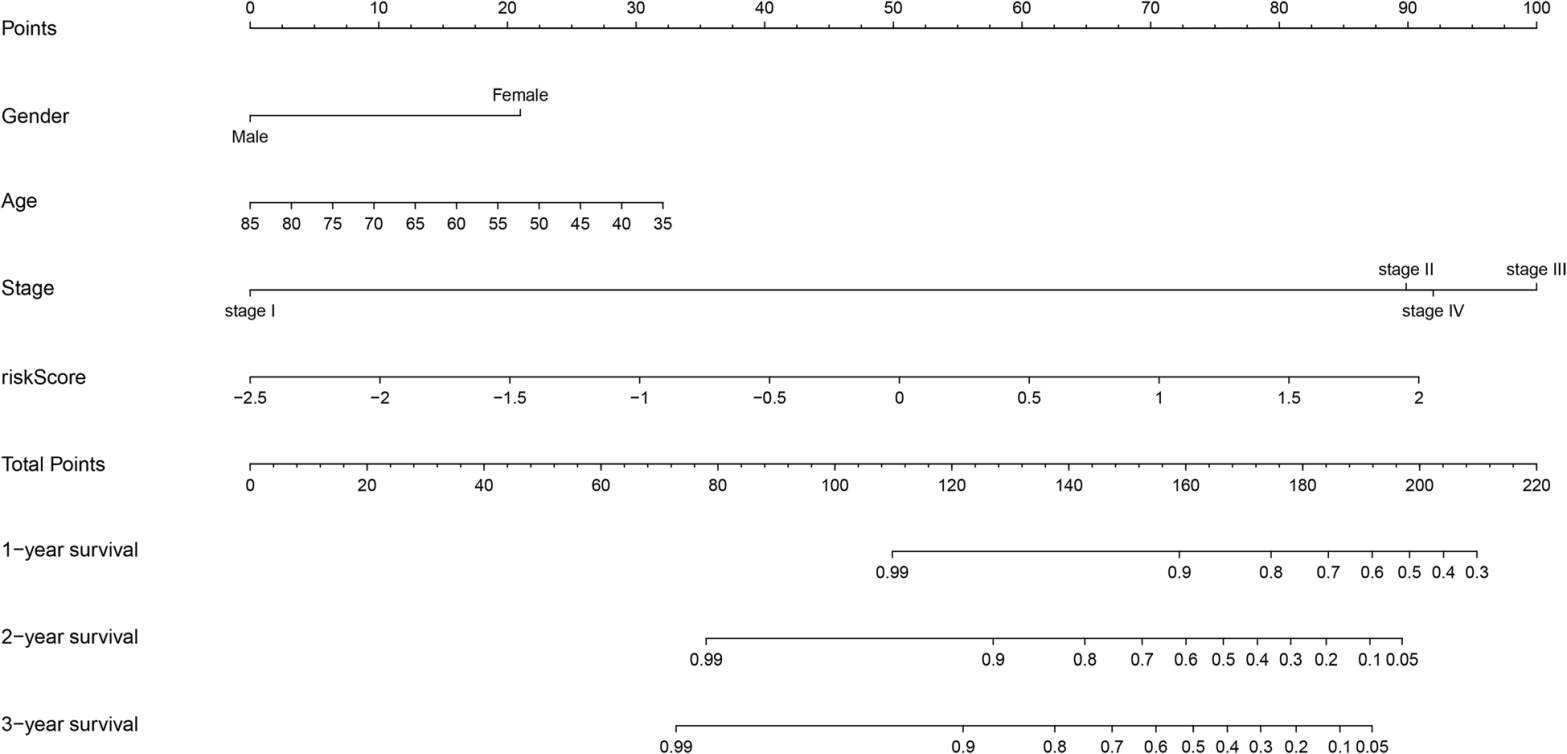
A nomogram to predict survival probability at 1, 2 and 3 years after surgery for laryngeal cancer patients, based on the results derived from the entire set

Univariate and multivariate Cox regression analyses were used to investigate whether the 4-lncRNA signature was an independent predictor of prognosis among laryngeal cancer patients, using the risk score and other clinicopathological data, including age, sex, and stage. In the univariate Cox regression, the risk score and sex were significantly correlated with patients’ overall survival. In the multivariate Cox regression analysis, after adjusting for age and other covariates, the risk score based on the 4-lncRNA signature remained an independent predictor (Table 3). Moreover, a nomogram was constructed that integrated the 4-lncRNA signature risk score and other clinicopathological characteristics such as age, sex, and stage. A higher total number of points on the nomogram indicates a worse prognosis (Fig. 6).

**Table 3:** Univariate and multivariable Cox proportional-hazards regression analysis on OS

Univariate Cox				multivariate Cox		
Variables	HR	95% IC of HR	*P-*value	HR	95% IC of HR	*P-*value
Sex	0.30	0.132–0.701	0.005	0.376	0.154–0.917	0.032
Age	1.00	0.959–1.046	0.935	0.972	0.931–1.014	0.190
Stage	0.91	0.560–1.466	0.687	1.024	0.606–1.731	0.929
riskScore	2.39	1.698–3.370	0.000	2.522	1.727–3.685	0.000

### Prediction of lncRNA-related genes and functional annotation

**Fig. 7:**
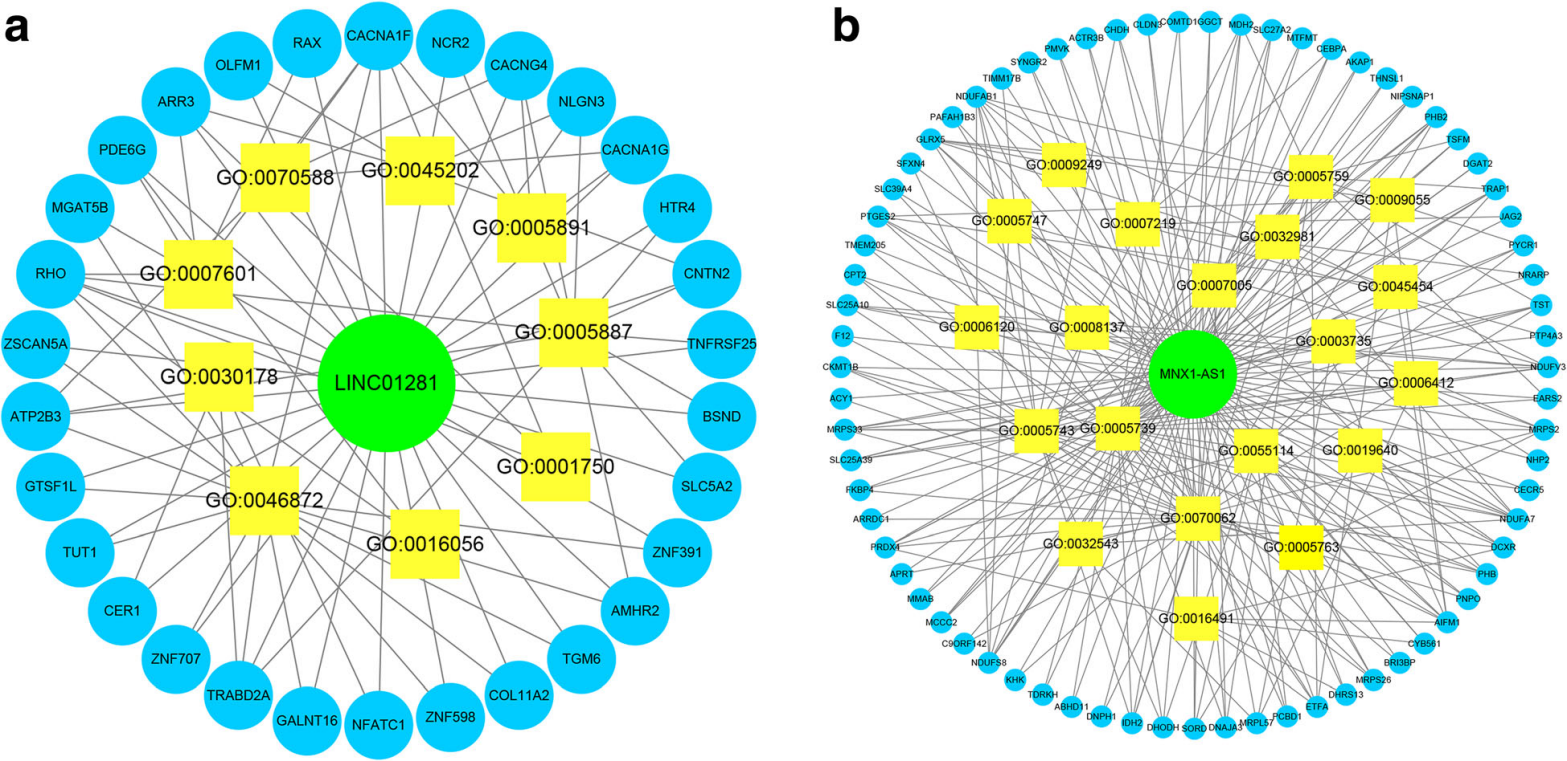
Relationship between the lncRNAs and related genes, and GO functional annotation analysis results. **a** The related genes of LINC01281 and the GO functions that they enriched on. **b** The related genes of MNX1-AS1 and the GO functions that they enriched on

We identified genes related to the signature lncRNAs using the MEM database. The top 200 genes that most closely related to LINC01281 and MNX1-AS1 are listed in Additional file 3. We were unable to search for genes related to LINC02154 and MYHAS in MEM. The relationship between the lncRNAs and the related genes and the GO functional annotation analysis results are shown in Fig. 7.

## Discussion

Laryngeal cancer is a malignant head and neck squamous cell carcinoma [18]. Currently, several therapeutic strategies such as surgery, radiation therapy and chemotherapy are employed in clinical treatment. Owing to the development of treatment strategies for this malignancy, the survival rates of laryngeal cancer patients have improved to some extent [19]. However, the prognosis of patients with advanced laryngeal cancer has not improved over the past several decades. In fact, metastasis and recurrence are the main causes of poor prognosis and decrease the overall survival of laryngeal cancer patients by more than 50% [20]. Because of the non-specific symptoms of laryngeal cancer, the disease is often diagnosed late, when it is already in advanced stages, resulting in delayed treatment [21]. Therefore, there is an urgent need to explore novel and effective prognostic indicators in laryngeal cancer. The current development of bioinformatics technologies provides powerful, high-throughput tools to screen molecular biomarkers and indicators of prognosis. Recently, increasingly more researchers have focused on the clinical significance of lncRNAs on the prognosis of multiple types of tumors; these lncRNAs have tissue-specific expression patterns and play crucial roles in the progression of diseases. For instance, Zhang K et al. [22] observed that the lncRNA AOC4P promotes tumourigenesis and progression partly through epithelial-mesenchymal transition, thereby promoting a poor prognosis in gastric cancer. The study by Bo H et al. [23] showed that the expression level of the lncRNA AFAP1-AS1 may be involved in the development of cervical cancer, and Beltrán-Anaya FO et al. [24] confirmed that the low expression of LncKLHDC7B was associated with poor prognosis in patients with breast cancer.

In our study, we constructed a 4-lncRNA risk model that predicts prognosis in laryngeal cancer. MYHAS and LINC01281 were associated with a better prognosis among laryngeal cancer patients. Therefore, this novel 4-lncRNA signature could be used to predict OS for laryngeal cancer patients. Furthermore, using the MEM database and functional enrichment analysis, we hypothesized possible mechanisms by which lncRNAs influence the prognosis of patients with laryngeal cancer. According to the functional annotation results, genes related to MNX1-AS1 significantly enrich on GO terms 0007219 (Notch signaling pathway) and 0070062 (extracellular exosome). The Notch signaling pathway is evolutionarily conserved in mammals and plays an important role in cell development and differentiation. In recent years, increasing evidence has shown that Notch is associated with tumor development. Aberrant activation of the Notch signaling pathway has been found in many different solid tumors, and it has been found to induce cell proliferation, metastasis and epithelial-mesenchymal transition [25]. The exosome has been found to play important roles in many physiological and pathological processes such as antigen presentation in immunization, tumor growth and migration, and repair of tissue damage. Exosomes secreted by various cells have different components and functions and they are used as biomarkers for disease diagnosis. Exosomes possess a lipid bilayer membrane structure that can protect the coated material well and can target specific cells or tissues, so they constitute a well-targeted delivery system [26]. Generally, the possible mechanisms by which MNX1-AS1 may lead to poor prognosis are mainly related to immune response and the Notch signaling pathway. LINC01281 enriched on GO terms 0005891 (voltage-gated calcium channel complex) and 0030178 (negative regulation of Wnt signaling pathway). Voltage-gated calcium channels (VGCCs) are well documented to play roles in cell proliferation, migration, and apoptosis. Chih-Yang Wang et al. showed that many calcium channel subunits are overexpressed and likely involved in the development of various types of cancers. The observed overexpression of CACNA1A, CACNA1C, and CACNA1D suggests that they are likely targets for cancer treatment, as blockage or partial inhibition of their expression could help to modulate the progression of metastatic disease [27]. Many previous reports revealed that the Wnt signaling pathway is a highly conserved pathway controlling cell growth, differentiation, apoptosis and self-renewal. This signaling pathway is often abnormally activated in tumor development and progression, and it can enhance or antagonize other signaling pathways to regulate tumor proliferation, migration, and invasion [28–30]. Based on our prediction and functional annotation analysis of genes related to our selected lncRNAs, we speculate that these lncRNAs may influence the prognosis of laryngeal cancer through mechanisms involving immune response, the Notch signaling pathway, voltage-gated calcium channels, and negative regulation of the Wnt signaling pathway.

In summary, we constructed a novel 4-lncRNA signature that can be used to predict the prognosis of patients with laryngeal cancer and hypothesized possible underlying mechanisms. However, there are some limitations in our study. First, we obtained data only from the TCGA datasets, and the sample size is not very large. Second, we provide only preliminary hypotheses on the possible mechanisms by which the selected lncRNAs impact prognosis. Further studies should be conducted to clarify the overall mechanism by which these lncRNAs influence the prognosis of laryngeal cancer. Finally, all of the above results need to be verified in basic experiments and clinical trials.

## Conclusion

We constructed a novel 4-lncRNA signature that can predict the prognosis of patients with laryngeal cancer. The mechanisms underlying this predictive relationship may involve the extracellular exosome, the Notch signaling pathway, voltage-gated calcium channels, and the Wnt signaling pathway. These findings may provide a better understanding of the prognostic value of lncRNAs and related mechanisms.

## Additional files

Additional file 1: ceRNA network. Competitive endogenous RNA (ceRNA) regulation network of differentially expressed lncRNAs in laryngeal cancer. (PDF 7834 kb)

Additional file 2: R language code. R language code of Multivariate Cox regression analysis to develop a 4-lncRNA signature. (TXT 1 kb)

Additional file 3: lncRNA-related genes. Top 200 lncRNA-related genes predicted by MEM. (PDF 7558 kb)

## Data Availability

The datasets supporting the results of this article are publicly available at the TCGA (https://portal.gdc.cancer.gov/).

## Abbreviations

AUC: area under the curve
GO: Gene ontology
KEGG: Kyoto Encyclopedia of Genes and Genomes
MEM: Multi Experiment Matrix
OS: Overall survival
ROC: receiver operating characteristic
TCGA: The Cancer Genome Atlas Database
