# Supplementary figures and images for: Identification and potential mechanisms of a 4-lncRNA signature that predicts prognosis in patients with laryngeal cancer
Source: Hum Genomics. 2019 Aug 15;13:36. doi: 10.1186/s40246-019-0230-6 (PMC6694645; doi:10.1186/s40246-019-0230-6)

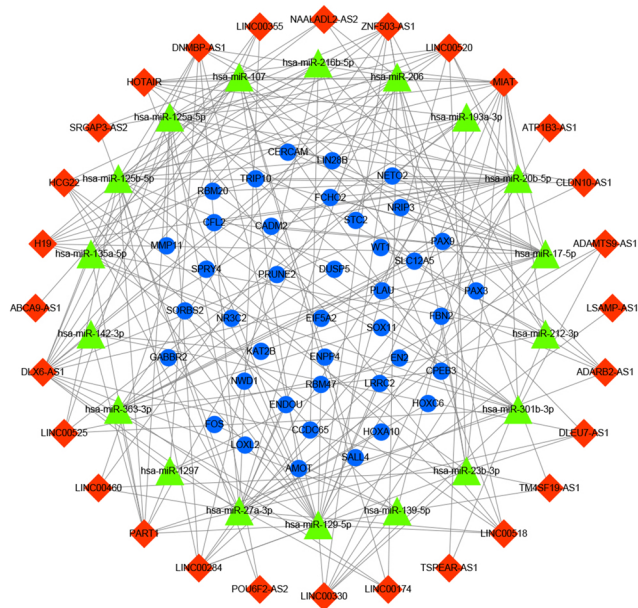

Supplement: Supplementary file 1 — ceRNA network. Competitive endogenous RNA (ceRNA) regulation network of differentially expressed lncRNAs in laryngeal cancer. (PDF 7834 kb) [file 40246_2019_230_MOESM1_ESM.pdf]

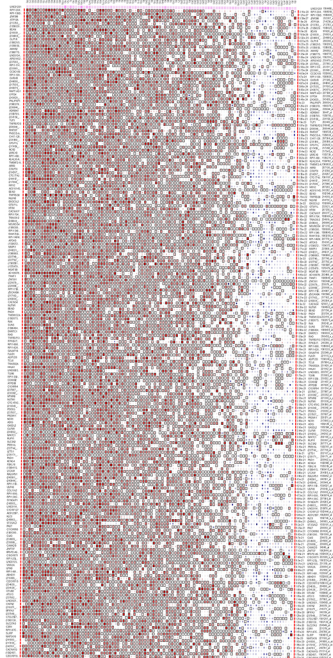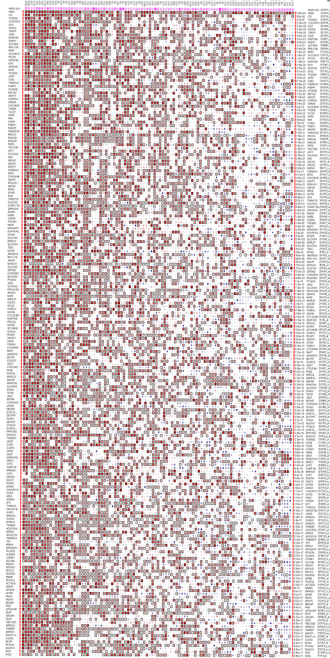

Supplement: Supplementary file 3 — lncRNA-related genes. Top 200 lncRNA-related genes predicted by MEM. (PDF 7558 kb) [file 40246_2019_230_MOESM3_ESM.pdf]
